# Exploring chronic pain related attentional experiences, distress and coping strategies among Arabic-speaking individuals in Jordan and the United Kingdom

**DOI:** 10.3389/fpsyg.2023.1268179

**Published:** 2023-10-02

**Authors:** Ahmad N. Abudoush, Khalifa Ali, Tayyeba Kiran, Maria Panagioti, Ellen Poliakoff, Nang Mo Hom, Nusrat Husain

**Affiliations:** ^1^School of Health Sciences, The University of Manchester, Manchester, United Kingdom; ^2^Department of Psychology, The University of Jordan, Amman, Jordan; ^3^Department of Research and Development, Pakistan Institute of Living and Learning, Karachi, Pakistan; ^4^Research Capacity and Capability Program, Ethnic Health Forum, Manchester, United Kingdom

**Keywords:** chronic pain, attention experiences, qualitative, framework analysis, Arabic, distress, coping strategies

## Abstract

**Introduction:**

The lived experiences of chronic pain (CP) among Arabic-speaking populations remain underexplored. A better understanding of these experiences and their associations with attention difficulties, coping mechanisms, and treatment options could lead to improved support for this group.

**Methods:**

This qualitative study utilised a descriptive design and involved one-to-one interviews with 51 participants with CP who had just completed two attention tasks. Interviews were conducted using a semi-structured topic guide, transcribed verbatim and translated from Arabic to English before agreeing on the coding framework. Themes and subthemes were extracted using a framework analysis approach.

**Results:**

The study identified six main themes: Factors contributing towards developing or exacerbating CP, the impact of CP on psychosocial functions, including attention, the perceived role of social support, coping strategies for managing CP, perceptions about available treatments and recommendations for interventions.

**Discussion:**

CP significantly impacts individuals’ physical and psychosocial functions, and it is reciprocally associated with attentional difficulties. Despite using various approaches to manage their CP, none of the participants used psychological interventions or counselling. Understanding the diverse impacts of CP and the coping strategies employed to develop culturally sensitive interventions, review current related policies, and improve healthcare services is crucial to managing CP among this population.

## 1. Background

Chronic pain (CP) affects more than 30% of people globally and hence is a major personal and economic burden (Cohen et al., 2021). Two CP conditions, namely neck pain and back pain, are among the top leading causes of years lost to disability (Vos et al., 2017). Studies among Arabic populations concur that the prevalence of CP is high (20–46.4%, Elzahaf et al., 2016; Almalki et al., 2019). CP can have a multifaceted impact on the health and well-being of individuals (Tanaka et al., 2022). Attention is one of the important factors that have been linked to CP (Diotaiuti et al., 2021). However, the focus of the previous literature has been the quantitative scaling of CP-related symptoms and examining its subtypes (MacNeela et al., 2015). Attention is typically explored using experimental tasks (Crombez et al., 2013). A recent systematic review and meta-analysis found that people with CP exhibit significant attentional bias toward sensory pain-related information, however, these experimental tasks have not been used previously among the Arabic population (Abudoush et al., 2023). Moreover, there is no qualitative research internationally to understand the subjective attentional experiences of individuals with CP, coping mechanisms, and potential treatment pathways. A qualitative exploration can balance out the limitations of the quantitative methods and provide a deep insight into the perspectives of the individuals on challenges accompanying CP and their views about possible interventions (Kelle, 2006; Ritchie et al., 2013).

Because of its chronic nature, coping with CP is a key component in understanding how positive or negative strategies are used on a daily basis (Dysvik et al., 2005). Autonomy-driven approaches encourage individuals with CP to be more independent and resilient, which helps active engagement in therapy and life (Gittell, 2016; Gorman-Badar, 2020). Furthermore, exploring the experiences of Arabic individuals with CP specifically can help to examine the sociocultural factors (e.g., social values, religious beliefs, and language) that might be specific to this population. The sociocultural perspective is important due to its multidimensional impact on shaping how individuals respond to, and cope with, their CP. Thus, considering a culturally sensitive approach, such a perspective can help understand how CP-related difficulties might be used in developing an attention-related intervention through patient-driven options. This study aimed to explore the attentional experiences, coping mechanisms and suggestions for treating CP among Arabic-speaking individuals in Jordan and the United Kingdom (UK).

## 2. Methodology

### 2.1. Design

A qualitative study nested within an experimental study. All the participants in the CP group (experimental group) of the study were invited to participate in the qualitative part of a larger empirical study (the original sample included 58 CP participants and 58 healthy controls).

### 2.2. Setting

This was a hybrid study (i.e., conducted remotely and in-person) with participants recruited from pain clinics, physiotherapy clinics, community centres, and hospitals from two study sites (i.e., Jordan and The United Kingdom). For complete information about the design, see the original experiment by Abudoush et al. (2021).

### 2.3. Eligibility criteria

#### 2.3.1. Inclusion criteria

Arabic-speaking individuals with CP (i.e., pain for 3 months or more) who were aged 18 years or above, have a normal or corrected to normal vision, have a native speaking, reading, and writing of the Arabic language, resident of either Jordan or the United Kingdom at the time of the experiment, can complete experimental tasks with 70% or more of accuracy assessments, have access to a laptop or desktop with an internet connection for at least 90 min for one time, willing to participate in the study, and can provide informed consent.

#### 2.3.2. Exclusion criteria

Individuals were excluded if they were having any severe or uncontrolled mental or medical disorders that would affect their participation and/or having a current acute or subacute pain.

#### 2.3.3. Description of the experiment

The experimental study was pre-registered on the open science framework (OSF; Abudoush et al., 2021). It involved exposure to pain-related words such as sensory-related (e.g., stabbing, pinching), and affect-related (e.g., punishing, frightful) words. Two groups (i.e., CP and matched healthy control) were tested for their selective attention performance using two experimental tasks (i.e., spatial cueing task, and Emotional Stroop task). Also, a number of questionnaires were used to assess participants` resilience, perceived stress, anxiety, and depression levels. The qualitative study aimed to explore the participants` subjective attention-related experiences that cannot be measured using quantitative methods.

### 2.4. Recruitment

The study was approved by the University of Manchester Research ethics committee number 3 (UREC 3) (2022-11074-21987) in the UK, and the Jordanian Ministry of Health (MoH) (Moh/REC/2021/233) in Jordan. A total of 116 participants (*N* = 58 in the CP group and *N* = 58 in the healthy control group) matched for age, gender and country of residence were recruited through online advertisements and posters at pain clinics, physiotherapy clinics, community centres, and hospitals. Interested potential participants directly contacted the researcher to participate and were screened against study eligibility criteria, followed by sending and explaining the participant information sheet (PIS). Participants received reimbursement for their time.

For the qualitative nested study, participants from the CP group who gave additional consent for the interview were included. All 58 individuals with CP who participated in the main experiment were invited for a one-to-one interview. Seven of them did not consent to participate. A total of 51 participants formed the final sample and completed the interview. Although there are no specific limits for the sample size in the qualitative interview, it is worth mentioning that our sample is considered within a reasonable sample size range (Dworkin, 2012). A reasonable sample size would ensure that all possible themes and subthemes are covered in the study and that there is no new data generated when conducting additional interviews. Dworkin (2012) suggests a sample size that ranges between 5 and 50 participants.

### 2.5. Data collection

A semi-structured interview was conducted directly after the experimental study. A semi-structured topic guide was developed to facilitate the interview. The topic guide aimed to explore four main areas to identify and elicit details on the following;

1) How their daily attentional experiences are affected by CP,
2) Attention experiences related to exposure to pain-related cues within the experimental tasks,
3) Participants’ perspectives on coping with CP, and
4) Participants’ views about possible interventions that could be of benefit.

The topic guide was developed by two authors (AA and TK) and reviewed by the other co-authors. AA is an expert in the CP-attention field, and TK is an expert in the qualitative research field (Appendix 1). To ensure the suitability of the questions included, the topic guide was reviewed and modified according to feedback from an individual with CP who was included in the study according to the patient and public involvement and engagement (PPIE) principles. It included a combination of open and closed questions to help participants express their emotions and thoughts related to the CP. The topic guide was also updated during the period of the data collection phase according to feedback from participants. The first section included questions about the daily attentional experiences affected by the CP, which is fundamental to understanding the impact of the CP on their attention, especially those related to the tasks that require attention. The second section explored the included the attention experiences related to the exposure to pain-related cues, which is crucial to understanding the effect of participating in an experimental study that contains pain-related cues in the Arabic population. This is essential for future research in this population. The third section included the different perspectives related to coping with CP, which is vital to understanding the patterns of adjusting to attention-related difficulties and what coping strategies might help in overcoming such difficulties. The last section in the topic guide was about the possible interventions, which explored the participants’ opinions about what could help them to overcome the challenges related to the CP and related attentional difficulties.

The interviews were conducted by the first author (AA), who is bilingual (i.e., Arabic and English), an experienced clinical psychologist who has worked with patients with CP for many years. All interviews were conducted using the Zoom application (Zoom Video Communications Inc, 2022), audio recorded and saved on a secure server until the transcription phase was done. All interviews were transcribed by two authors (AA and KA) and reviewed for accuracy. Initially, seven interview transcripts were translated into English and coded by (AA), and these coded transcripts were discussed with a senior qualitative researcher (TK). Both AA and TK agreed on an initial coding framework. For the remaining interviews, Arabic transcripts were coded using the coding framework by (AA), and only coded verbatims were translated from Arabic to English. To minimise the loss of meaning, the authors followed the recommendations of Van Nes et al. (2010). For example, the authors used fluid descriptions of meanings when necessary rather than relying only on the direct translation to allow for an accurate contextual meaning of the verbatims. The interview duration mean was 21 min, and interview time ranged between 11 and 47 min.

### 2.6. Analysis

An inductive framework thematic approach was used to analyse the results (Gale et al., 2013; Ritchie et al., 2013). The first author conducted the familiarisation stage (AA) and reiterated through transcripts to fully understand the data. Then, researchers independently coded seven interviews (AA and TK), discussed the codes resulting, and agreed on the framework themes and sub-themes. Next, the first author (AA) completed coding and categorising the other 44 interviews independently. Then, TK reviewed all interview codes and translated verbatims to ensure accurate coding, categorisation, and their dependability. A discussion between all authors was held to agree on the analytical decisions and then on the final codes matched to the verbatim list to ensure credibility and dependability.

To ensure the quality of the analysis and the reasonableness of the sample size, researchers considered the data saturation concept during the analysis process (Braun and Clarke, 2021). Further, because of the numerous subtypes of CP a large sample was essential to ensure that different aspects of CP-related experiences were covered. The resulting large data was managed by the researchers (AA and KA) under the supervision of a senior qualitative researcher (TK). Using framework analysis helped in organising such data in a structured way for easier interpretations. The suitability of analysis and confirmability was ensured through re-iteration of the transcripts, agreeing on the code book (i.e., framework identification), and regular review of the progress of data analysis. Further, despite the potential effect of the prior experience of the researcher on shaping the findings, researcher bias was avoided by ensuring that the different co-authors were involved in all steps and decisions related to data analysis.

## 3. Results

For demographic characteristics, all participants have Arabic ethnicity. We interviewed 51 participants, 33 of whom were based in Jordan and 18 in the UK. Thirty-one participants were male and 20 were female. The mean age of the participants was 42 years (SD = 14), and two-thirds were married. At least half of the participants had a University/college degree, and the vast majority (48 participants) had medium to high-income levels. The most common types of pain were low back pain (12 participants), headache (9 participants), and chronic primary musculoskeletal pain (9 participants). The average pain intensity (i.e., out of 10 points) between interviewed participants was moderate to severe (M = 5.67, SD = 2.07). The pain duration ranged between 4 months and 24 years (M = 7.21, SD = 6.53).

### 3.1. Framework analysis themes

**Figure 1:**
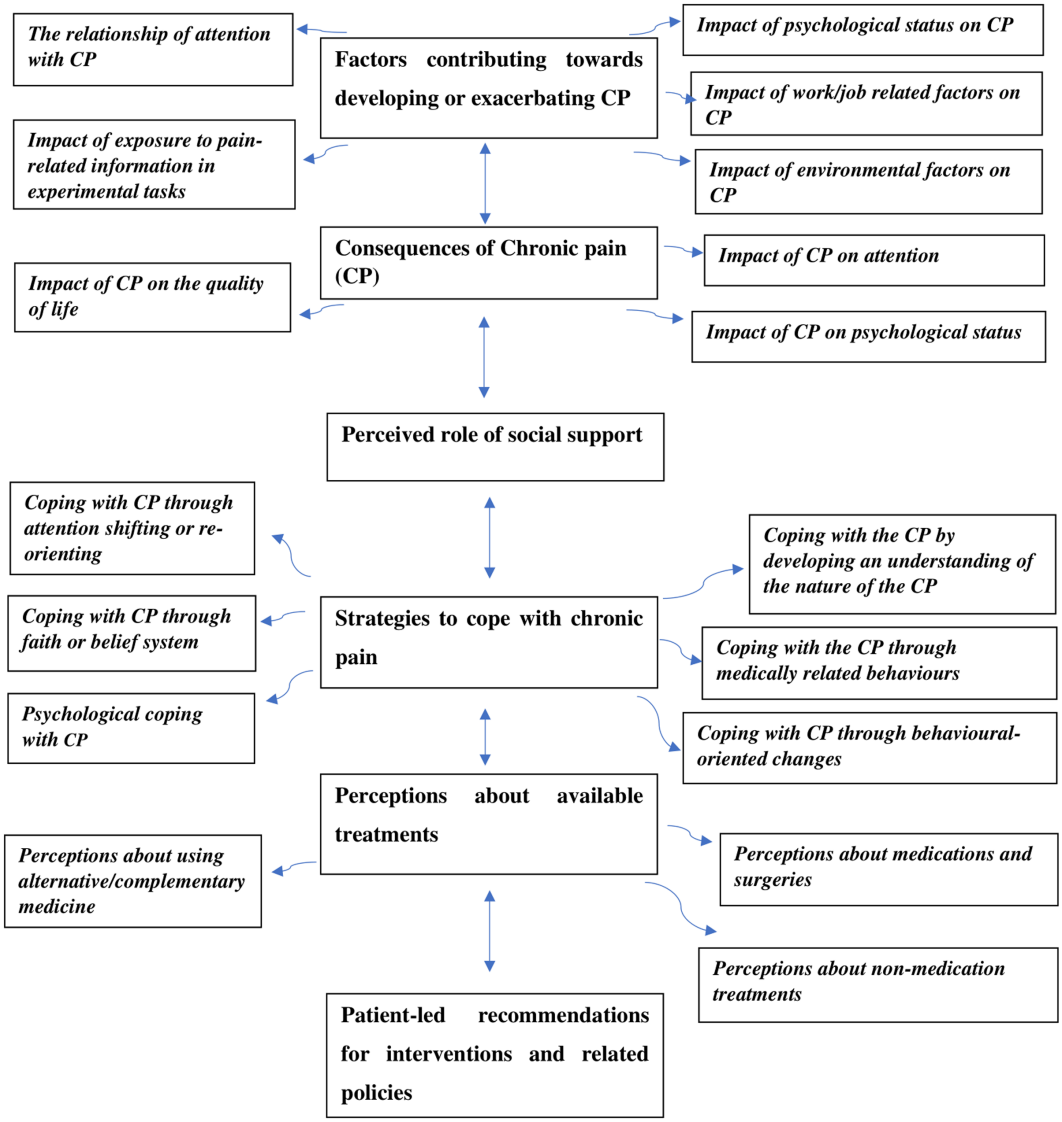
A diagram showing the themes and subthemes.

A framework was created using Microsoft Office Excel sheets comprising 51 interviews and six main themes representing the study’s objectives around lived experiences of CP, reciprocal impact between CP and attention on daily attentional experiences and exposure to pain cues, available treatments and perspectives about coping strategies with CP (Figure 1). An example of the process of going from meaning to formulating themes is illustrated in Table 1.

**Table 1:** An example of the process of going from meaning unit to code to category to sub-them to theme.

Meaning Unit	Code	Category	Sub-theme	Theme
*“If pain is severe, I have to stop doing the task because it affects my concentration ability”* (P2)	Impact on attention when the pain is severe	Factors contributing towards developing or exacerbating attention difficulties	Impact of CP on attention	Consequences of Chronic pain (CP)
*“I am (Having) attention because I am too arranging things and know how to manage it. Attention and focus will improve, and attention and focus are (good) from a lot of what I am (doing). On the contrary, (because) I am paying attention and concentrating it made my illness improve.”* (P42)	Coping through arranging	Coping with CP through reorienting	Coping with CP through attention shifting or re-orienting	Strategies to cope with chronic pain

#### 3.1.1. Factors contributing towards developing or exacerbating CP

This theme emerged from the first and second topic guide points. Different factors were identified as contributing to developing, exacerbating, or maintaining CP among Arabic-speaking individuals. These factors were explained through five subthemes.

##### 3.1.1.1. The impact of psychological status on CP

Participants perceived that the severity levels of CP were related to their psychological conditions. A couple of participants from both study sites highlighted the connection between their mood and the pain they experienced, and one participant shared as

“*I feel that if I’m emotionally tired and I have pain, the pain can also increase, but if I have pain and my mental health is good, the pain does not decrease, but I think of it less.”* (P44)

Many participants firmly stated that the pain intensity went in tandem with stress at home or work. Psychosocial pressure, such as stress at work, the tension of assignments, and family pressure, intensified the pain. One female participant reported;

“*If I increased my concentration more than what was needed, the pain increased because I would have been in stress, but if my concentration was normal and moderate, which is a regular day, for example, that you work during the day in the normal hours, so that is fine.*” (P49)

##### 3.1.1.2. The impact of work/job related factors on CP

Work behaviours were one of the sources that exacerbated the CP. Nature of work, such as *sedentary* (P43), or having worked in a prolonged fixed position, e.g., *long screen time in one position* (P35), or *excessive standing* (P32), had an effect on the degree of pain suffered. Participants realized their pain increased with *specific postures at work, mainly when they had a deadline* (P49). One female student participant expressed how her routine at the study workstation was connected to the pain.

*“I feel that the pain increases when I work a lot on a computer or when I am stressed, I mean, lots of things to do on that day. It is not a requirement that it be related to study, (but) it means that it is related to my normal life. You know, this is what happens when I feel that it is a period of tension, a period of pressure.”* (P1)

##### 3.1.1.3. The impact of environmental factors on CP

Participants identified that lifestyle factors such as diet, obesity, *“not exposing enough to sunlight”* (P3), *“cold weather”* (P13), and *“lifestyle stress”* (P12) had an effect on CP. Food quality or the type of food influenced the development of CP in a few participants. Some kinds of food, for instance, ready-made food, were perceived as inappropriate for their illness, and that *“food is not giving benefit”* (P3), which made their pain worsened. Therefore, they tried to follow a healthy diet. For one particular housewife, *“weight gain”* (P42) made her CP persistent.

##### 3.1.1.4. The relationship of attention with CP

The relationship of attention with CP is consistent with the notion that focusing on pain intensifies it, whereas diverting attention towards something else decreases the pain (P12), and one participant described it as a “*paradoxical effect*” (P17):

*“I continue feeling the pain when I focus on the pain, I mean if I did not distract myself (to something else).”* (P11)

Participants also mentioned other ways in which attention can contribute to exacerbating pain. They reported that tasks requiring greater attention could increase pain intensity. Pain became noticeable when they focused on specific functions, e.g., reading or working at home. This experience was also related to body posture while concentrating on computer-related tasks. Student participant experienced it and shared:

*“When I am concentrating on something, or I have something, the pain increases, yeah, exactly it increases if I read or study or work at home, all of these increase the pain”.* (P16)

##### 3.1.1.5. The impact of exposure to pain-related information in experimental tasks

In the context of the experiment that preceded the qualitative interviews, participants were requested to respond as soon as possible to target information (i.e., degree of the colour of a green frame surrounding a black square in the Posner spatial cueing task, and the colour of the word in the emotional Stroop task). The target information appeared after or during presenting pain-related cue. In their reflections on this experiment, some participants highlighted that conducting attention experimental tasks needed concentration and extra effort to keep focusing on the task, which made them a *“little exhausted”* (P13). One participant explained the pain-related information:

*“It is possible some words for me, they are, as I told you, big. For example, such as kicking out and stabbed.”* (P42)

Nevertheless, other participants saw this attentional exposure as a source of *“excitement”* (P1) or as *“brainstorming*” (P9). One participant explained the experience of attention during the experimental task as follows:

*“If I focus on the thing that they told you to, on the cross, you will find it easy, great, if I start to oversight and look at the words, I forgot once or twice, and so I realised that I got mixed up…if I focused on the information that I have, the cross sign, and focus on the colours and answer fast with focus, but if you scattered the words you can’t.”* (P32)

Positive feedback on the experiment was also reported by the participants. Participants highlighted that the activities included in the task were enjoyable. The *“rehearsal”* (P42) part helped them to be prepared for exposure to pain-related information and completing the experimental tasks. One male participant highlighted that the good thing about the attention tasks was that they *“made one reflect on themselves in relation to the topic of attention, sometimes we do tasks, or we carry out tasks without concentrating or with partial concentration.”* (P50)

#### 3.1.2. Consequences of chronic pain

This theme emerged from the first topic guide point. Three subthemes that explain the impact of CP were identified from the interviews as follows:

##### 3.1.2.1. The impact of CP on attention

The impact of CP on attention was frequently mentioned in different forms, including *“If pain is severe, I have to stop doing the task because it affects my concentration ability”* (P2) and that *“focusing tires and exhausts me when I am in pain”* (P20), and *“it distracts my attention”* (P21). Interestingly, some participants linked the severity of pain (P49) with the attention they had throughout the time of the day, which one participant reported as follows:

“*It depends; sometimes, when the pain is severe, my concentration becomes scattered depending on the degree of pain during the morning, noon and afternoon. In the afternoon, the pain is very intense.”* (P23)

Moreover, the interruption caused by CP on attention can be seen in different dimensions of life. The pain made it difficult for the participants to focus on their surroundings or work. They were required to put more effort when they tried to remain focused on what they were doing, such as communicating with others (P50). One participant shared his experience:

*“All of your focus is on your pain, so you are not conscious about what is happening around you, like, when you are in a certain gathering, you will not be aware of some things there, who is there, who is praying, who is working, what is required from you. All your concentration, body and senses are focused on the pain.”* (P33)

##### 3.1.2.2. Impact of CP on psychological status

Impact of CP on psychological status was evident among some participants, such as CP perceived as “*distressing*” (P26) and “*psychologically irritating that increases negativity”* (P31). Participants were usually angry and frustrated (P19) as they had to live with CP, under pressure (P18), or feeling trapped, and they perceived that they could not have a normal life (P20). One participant reported;

*“Some days, you could find that I stopped bearing it (pain); I become nervous from it. I get angry that way, and why (I have pain)? I want to get rid of it, I want to get out of this thing. I want to live like any other normal person.”* (P12)

Participants mentioned pain as being associated with suffering, and their experience of CP created pessimistic views. Most of them explicitly described how CP negatively affected their mental status, such as *desperation* (P25), *hopelessness, disability* (P45), *having a bad mood or being secluded* (P36):

“*When I get a pain episode, I get isolated, and I feel the world is black. It causes me much sadness. Then depression creates (further) depression. Yeah, sometimes I cry, become nervous, and tense. I isolate myself to avoid getting anyone angry. It’s the hardest thing, I get a psychological condition when I got a pain episode.”* (P10)

A couple of participants expressed the fear and being *“careful and scared”* (P22) related to the CP. They were fearful or anxious about escalating CP (P8) or of developing imminent pain in other parts of the body. One participant also shared her worries about getting a proper diagnosis, so she tried to procrastinate seeking medical checks. Also, self-blaming and *“internal conflict”* (P50) arose after the pain had been exacerbated.

“*I am living with the illness, but I still have that fear that it will increase, that it will spread to my hands. I remain scared because it is in the neck and shoulders to radiate down to my hands. The fear, the worry. I am scared that it will spread to my hands.*” (P11)

##### 3.1.2.3. The impact of CP on the quality of life

The Impact of CP on the quality of life was described as having a *“great negative effect”* (P22). Participants reflected on the chronicity of pain through the necessity for “*adapting to the situation*.” Some participants were *forced to give up what they liked* (P39) and *to navigate life without having any (self) control* (P42). They became dependent and relied heavily on their families or friends, even for certain routine chores such as taking out clothes from the wardrobe or getting a sofa (P50). Participants compared their life before and after having the CP, and one of them reported:

*“It influences everything, sleep, work, studies, everything, like, it’s tiring. That’s it your head hurts, you become unable to do anything like before.”* (P36)

Pain also limited the participants’ quality time, mainly with family; they became secluded and pushed away family members to avoid being seen in pain or tried to avoid recreation. Some participants also explained how CP negatively affected their social relationships and social engagement. Under the effect of pain, they could not interact with others, leading them to *“less participation in society”* or *“avoid people”* (P42) eventually.

Participants with CP also suffered sleep disturbance or lack of quality sleep (P12). They repeatedly woke up during the night due to pain, which was even harder for participants with certain types of CP, such as chronic back pain (P43) or in patients whose symptoms got worse at night time (P38), and sometimes, they required sleeping pills.

Participants also faced *“challenges”* (P2) in daily life due to CP, including *“work”* (P18), *“house chores”* (P18), *“praying”* (P19), “*studying”* (P21), *“cooking”* (41), *“driving”* (P45), and *travelling far distance”* (P50). Some participants described this impact as an *“actual disability”* (P9) that required staying home for a long time.

#### 3.1.3. Perceived role of social support

This theme emerged from the third topic guide point related to coping with CP. Participants with CP acknowledged the positive role of having social support and being treated with empathy. Many participants regarded families, friends, colleagues, and neighbours as very helpful in terms of soothing the pain or even helping distract their attention from the pain. The psychological aspect of treatment was considered more important than painkillers or other medical-related interventions, and social support could represent *“collaboration, cooperation and sensitivity”* (P31). A few participants also shared their experience of receiving positive psychosocial support during severe pain episodes. The role of family support was explained by one participant:

*“The most important thing is the family, like, this affects me very very much from a psychological point of view and gives me, like, a push to keep going, like, how should I say this, positivity in my mental health. I feel much better emotionally when you have people around you, your family.*” (P40)

Participants perceived the *encouraging and caring words* (P14) as mental support which made them feel compassion and security. They particularly mentioned that *chatting* with someone, including colleagues or friends (P48), reduced their pain intensity and made them more comfortable. They greatly acknowledged the power of words from others such as *“do not give up, resist, try moving”* (P10). Some viewed social support as an essential key element in pain management, and other treatments came later (P33). A participant enthusiastically shared as;

*“The relief is more psychological than if it is physical. The second thing is it possible, just because the people around me, I mean, social support in general, they talk to me, I mean, they ask me about my pain and if I am getting better, oh, it helps, I mean, I don't know how to say it to you, but I mean, I feel a connection, I mean the social (bonds).”* (P1)

One important distinction participants made is that social support is not the mere number of people around them but the *quality of support* they receive from them (P42). Support by people who also experienced pain was also reported as beneficial.

However, a few participants also mentioned that *too much involvement from family members made them feel uncomfortable* (P13).

#### 3.1.4. Strategies to cope with chronic pain

This theme emerged from the third topic guide point. Participants shared their coping strategies related to managing their CP, which resulted in six main subthemes.

##### 3.1.4.1. Coping with the CP by developing an understanding of the nature of the CP

Some participants developed a detailed “*understanding of the nature of CP”* (P6), the *“reasons*” (P51) behind its persistence and coping strategies to achieve maximum functionality and minimise the impact of pain.

One participant reported:

*“(through) the person himself and his mentality and his understanding can know the nature of the pain, and the concept of creating pain inside the human and how to control this pain and cope with it, this has a huge role in human (life)…Let's say your closest friend, would you harm your closest friend? The same thing with pain.*” (P5)

##### 3.1.4.2. Coping with the CP through medically related behaviours

Aligning with the previous theme on the perceptions about the available treatment options, an overlap has been found with the coping strategies, where participants tried various different treatments to manage their CP with some participants trying several different approaches. These options included *“Physiotherapy” (P1, 11, 12, 14), “vitamins” (P4), “muscle relaxants and neck collar” (P11), “injections to reduce pain” (P9), “painkillers” (P15), “medical corset and creams” (P17), “painkillers and sleeping pills” (P21), “medications” (P30), “comfortable (medical) shoe” (P32), eyeglasses” (P34).* Participants also did some actions such as *“going to the hospital” (P9), “going to the doctor (P31), “following the instructions that could increase the back pain” (P33), “going to chiropractic to check the body alignment” (P48).* However, none of the participants tried psychological therapies to manage the CP.

##### 3.1.4.3. Coping with CP through behavioural-oriented changes

Participants used a range of behavioural activities to cope with existing CP, including physical activities such as *“sports exercises”* (P47), *“swimming”* (P32), *“going to the gym”* (P48), *“immediately stopping what I am doing”* (P1), *“go to a warm swimming pool”* (P50) and *“walking when the sun is out”* (P4). Other participants tried to change the way of performing tasks to find a “*better and easier”* (P2) way, such as *“monitoring”* their pain location (P20). Coping through a *“changing lifestyle”* (P42) and daily routine were reported as strategies that help keep pain at minimal levels, as well as following a *“healthy diet” (P3) or “drinking herbs and ginger” (P5).*

Other participants related coping through organising their time, *“doing everything in chunks”* (P14), *“following a programme every week”* (P17) and having *“enough sleep”* (P24). Work-related behaviours included being cautious when doing work tasks so that it would not affect their injury negatively. Two participants reported that they tended to change their posture while working or reduce the working hours to avoid exacerbating the pain. One middle-aged female reported her coping behaviours as follows.

*“Searching about any article or video and listening to my body… listens to something that relaxes them whether it is Qur'an or music calmness somewhat can relax, reduce the pain, yes, turning the lighting for example, like, if one takes a relaxation session.”* (P7)

##### 3.1.4.4. Coping with CP through attention shifting or re-orienting

Participants shifted their attention away from pain, such as changing the task they were doing or *“distracting”* (P10) themselves from pain by *“ignoring the pain”* (P27). To compensate, participants engage in other activities that *“preoccupy*” (P25) their attention and keep themselves *“busy as much as possible”* (P33), *“working on something useful, sat down and working or reading help forgetting or not forgetting but paying less attention to pain, overlook pain”* (P40).

Participants were trying in their daily life activities to focus on other more functional tasks, which, in turn, makes *“the sense of pain becomes lighter”* (P1). Some participants reported the reciprocal effect of taking care and attention to what a person should do and then ignoring the pain itself. One participant summarised this attention reprioritisation experience as follows:

*“I am (Having) attention because I am too arranging things and know how to manage it. Attention and focus will improve, and attention and focus are (good) from a lot of what I am (doing). On the contrary, (because) I am paying attention and concentrating it made my illness improve.”* (P42)

##### 3.1.4.5. Coping with CP through faith or belief system

Some participants mentioned their faith or belief as a source of “hope” (P45), which kept them “*positive and hopeful, and being patient”* (P2) and that supplication and *“spirituality”* (*P13) helped* to relax them (P13). Others saw the CP as a *“test”* (P5) that they must live with it. Some faith-related behaviours included reading *“the Qur’an, or doing Tasbeeh (remembering god)” (P10), “supplicates a lot” (P26), or “pray to Allah that he calms me” (P20).*

##### 3.1.4.6. Psychological coping with CP

Some participants focused on optimism and resilience-related mentality to adjust to the CP experience. Using these psychological resources to *“pay attention to the positive things”* (P42) and increase acceptance was an essential feature in some participants’ perspectives as they reported *“attempting to accept the suffering*” (37) and “*adapting to pain*” (P40). However, other participants highlighted the need to normalise pain, such as *“act as if I do not have pain*” (P29), and *“not giving up”* (P20). Further, participants mentioned the importance of motivating and *“energising”* (P3) themselves by *“remaining encouraged”* (P8). This positive perspective and persistence, in turn, helped some participants to preserve their attention while doing different tasks. One male participant reported:

*“I do not let anything prevent me from focusing on anything I do, whether it is simple or complex.”* (P46)

Participants reported that *“psychological readiness”* (P51) is essential in dealing with CP and that it is a crucial factor, so *“the first thing is to do something that will lift your mood”* (P29) because “*good mental health and that’s it*” (P21). One male explained that accepting that this is a chronic condition and dealing with it with a positive attitude is crucial:

*“I cannot refuse, I cannot change what happened, but I can change what is about to come.”* (P8)

#### 3.1.5. Perceptions about available treatments

This theme emerged from the fourth topic guide point. Three subthemes were identified explaining perceptions related to available treatments.

##### 3.1.5.1. Perceptions about medications and surgeries

Participants had varied experiences and opinions about medications, which is one of the first-line interventions for dealing with CP. Some participants perceived that oral medicines significantly reduced CP, and therefore, they felt comfortable (P24). Nonetheless, participants perceived that the effect of some medications notably decreased over time, and they were required to switch to another type (P41). One participant reported:

“*I tried painkillers that are Paracetamol exclusively. it is the only one that works for me. After many years of using it, its effect has diminished a little.*” (P46)

Most participants used different treatments to relieve the symptoms, including oral medicines, topical applications, injections, and surgery. For some participants, using one (e.g., pain killer alone) or a combination of medicines (e.g., muscle relaxants and injections) was effective and they had *a positive perspective on medications* (P6). However, some participants thought *medications produced only temporary pain relief* (P38). One young participant reported:

*“CP medications merely cure the symptoms, not the underlying reason.”* (P37)

Some participants reported being unable to adhere to medications due to fears of side effects in the long term. One participant reported:

*“Surely it (overuse of CP medications) will multiply diseases. I mean, for me, the medicine, in particular the medicine, I mean, I do not take it as a patient I have at home, does not take it, but only with caution. There is a need for someone (to supervise), and there is a need for a specific time for it (the medication).”* (P12)

Many participants were pessimistic about available treatments. They “*took the pain medication because of its availability*” (P33), and “*to manage and prevent (pain episodes) temporarily*” (P19) “*rather than cure the pain*” (P27). One participant reported:

“*There is nothing that I have come across that has helped me as I've felt.*” (P18)

Few participants did not like the idea of *“going to the hospital or clinic”* (P8) regularly for pain management and possible side effects. One participant explained:

*“If one continues to use these (medications), it can have an effect negatively on your organs in your body. So this is what, uh, in my opinion, has, to be honest, one should stay away from them as much as possible.”* (P3)

In addition to their side effects (e.g., drowsiness, kidney problems, liver problems, stomach aches), some medicines for CP were also considered expensive, which usually frustrated them. One participant noted:

*“Very tiring (the treatments). Uh really, really tiring and annoying and expensive. And I mean you get the frustration, I mean, I can't take (medication) every day. The problem of medications also is that it has side effects.”* (P10)

##### 3.1.5.2. Perceptions about non-medication treatments

Participants mentioned using non-medication treatments such as herbs and physiotherapy. Participants’ experience with physiotherapy was positive. They used physiotherapy in conjunction with other types of treatment, mainly medications. Participants felt comfortable while doing the exercises, and they perceived it was helpful in subsidising pain and reducing discomfort. Some perceived that physio-related medical equipment such as “*back belt*” (P4), “*leg band”* (P17), “*bandage*” (P44) “*warm device*” (P50) was beneficial. One participant reported:

*“I must strengthen my muscles, so the exercises are important, ice combined with the physiotherapy was better than taking medicines.*” (P47)

They also appreciated the specialist treatment of physiotherapists because they could do exercises with appropriate techniques, especially when they did their sessions with a physiotherapist. Some provided reasons for not following home physiotherapy as *“unable to manage their time at home”* (P43) or worried of worsening pain while doing exercises themselves. One participant revealed:

“*Physiotherapy just requires a long time because when I come home I get the pain, now it moves my fingers fine and lowers them well, but when I come home and I get the pain. I stop, I say to myself that I don't want (to do exercises), because I don’t want to be in more pain. So I let myself like this, just when I go to her (physiotherapist) she will work instead of me and I can tolerate that.”* (P41)

A few participants complained that the benefits of physiotherapy were temporary and that their pain returned after one to two sessions. Other participants differentiated between physiotherapy and exercises. For them, physiotherapy was not effective but the exercises, stretching and carrying heavy things and resistance training were extremely helpful in reducing the pain. Despite mentioning these treatments, none of the participants had undergone any type of psychotherapy or counselling for managing their CP.

##### 3.1.5.3. Perceptions about using alternative/complementary medicine

A few participants had tried various alternative/complementary medicine to relieve CP, including *“Chinese needles*” (P1), “*Arabic medicine*” (P5), “*herbs,”* and *“cupping”* (P6). Their belief in religion, together with alternative/complementary medicine had a positive effect on the pain management although they did not last for long. However, one participant regretfully shared his negative experience with alternative/complementary medicine since he perceived that the treatment could have ended up with him being in paralysis.

*“A month ago, I lost hope that I could go back to walking normally again or doing my daily routine. I tried a (traditional) prescription, and others suggested prescriptions for more than a month. I tried two or three (traditional mixes), and also I did not get any benefit. They took me to a person who treats using traditional Arabic medicine for that. I also didn't benefit. I benefitted one day, I mean, let’s say when he cracked (my back), he almost paralysed me.”* (P9)

#### 3.1.6. Patient-led recommendations for interventions and related policies

This theme emerged from the fourth topic guide point. Participants provided recommendations related to their physical activity for managing the CP. These suggestions focused on outdoor activities such as doing *“sports”* (P12) or *“going on a trip”* (P42). Other participants gave suggestions related to the faith and belief system. Some participants suggested that having spiritual life or religion would enhance *“inner peace”* (P6). Treatment-related suggestions to manage CP varied among participants, but they agreed that awareness of the factors that maintain the CP symptoms and *“understanding the reasons”* (P6) behind CP would be important in being *“self-sufficient”* (P1). A common piece of advice from participants was to seek proper medical treatment and commit to it. However, one participant warned that individuals should avoid excessive use of *“heavy painkillers”* (P30) and seek other options.

Adaptation to CP was one of the leading suggestions for managing CP through psychological willpower. Some participants thought that being able to *“adapt”* (P41) and *“tolerate pain endurance”* (P30) are essential to have a well-balanced life and keeping motivated for future planning. Interestingly, some participants linked hope to *“making others who also have pain happy”* (P6). One male highlighted the role of psychological status as *“You can enhance your psychological potential so that you are satisfied with the situation that you are in so you can continue with life”* (P31).

Participants agreed that the quality of services they receive from the health systems has a major impact on their well-being. The importance of increasing awareness about CP interventions. For example, via media was mentioned by one participant through “*the role of TV and advertising” (P6).* Some participants highlighted the need to involve policy-makers, media, and other influencing parties *to “support the use of recognised methods for pain management”* (P5) and provide proper *“training for professionals”* (P37). One older male highlighted (with a frustrated tone) the lack of support he received to manage his CP and the necessity to improve the services given to this population as;

“*There has to be special care. it is the right of the people by the government, that it takes care of their matters and provides them with services via their power, ability and their various ministries or specialities, ……….They should not leave people like on margin, and in their very limited financial situation, suffering until they lose their life, they have to, uh, care for us and maintain our dignity and provide us with everything that we need…… we need their ability to recruit all possible resources to cease suffering. ……you go to the hospitals and find them to be overcrowded, both in the outpatients and inpatients.*” (P31)

## 4. Discussion

This study explored the views of Arabic-speaking individuals on the impact of CP on their lives, attentional experiences, coping strategies, available treatments and CP daily management, as well as recommendations for future interventions to improve the management of CP. Factors contributing towards developing or exacerbating CP varied and included psychological (i.e., mood disturbance), contextual (i.e., job/work requiring attention) and social factors (i.e., social support). These findings align with previous studies which emphasised the link between CP and mental well-being (Turk and Okifuji, 2002). While some participants reported positive affect despite the CP, others had negative affect that impaired their functionality. Previous literature highlights that negative affect is the most assessed psychological factor associated with CP (Meints and Edwards, 2018), and that the pain tolerance of individuals with CP is low when their mood is low (Tang et al., 2008). Evidence also exists on the association between the availability of social support and improvement in CP symptoms (Turk et al., 2016). Most participants in this study preferred having supportive people around them, however, some participants preferred to be left alone. Having people in their close circle helped most participants manage their CP and maintain their optimism. It has been seen as a source of motivation to keep going in life.

Despite not being explored in the context of attention-related experiments before, the role of attention in exacerbating CP was clearly observed. Participants highlighted that focusing on pain (whereas distraction was a solution) as well as tasks requiring attention (such as performing experimental tasks during an experiment) were exacerbating factors. They also described difficulties in focusing their attention or concentrating when their pain was severe. A recent systematic review has highlighted that individuals experiencing CP find it difficult to complete tasks that require attention, such as driving (Vaezipour et al., 2022). However, the experience of being exposed to pain-related words during the attention experiment was acceptable for most of the participants, which reflects the safety of such exposure. This suggests that interventions such as attention bias modification (ABM) training, which is an attention training that enhances participants’ focus on the positive cues and away from pain-related cues, are likely to be acceptable to this group (Carleton et al., 2020). ABM training can potentially help in managing CP by reprioritising attention away from cues in the surrounding environment that trigger or maintain pain (Carleton et al., 2020).

In terms of impact, CP, made some participants feel hopeless with self-defeated opinions and behaviours related to suffering for a long period. They felt “forced” to live with pain, but the suffering is “optional,” as reported, so the psychological situation differs significantly. In addition to the psychological impact, CP-related attentional difficulties affected other practical aspects of the participants’ lives, such as not being able to concentrate on their daily tasks and minimised their productivity, which aligns with previous literature (Graziosi et al., 2022). These, in turn, adversely affected the physical and psychological health of people with CP (Kawai et al., 2017). There is established evidence of the impact of CP on the overall quality of life of this population, including physical functioning, and interference with professional life (Hadi et al., 2019).

Participants mentioned several coping strategies and most were able to adjust their lifestyles and manage their CP. Also, they expressed their perceptions about their ability to cope with CP in the future. However, some participants were unable to manage their CP, either because of a lack of awareness or high severity of pain; they tended to focus on pain and could not reorient their attention to other functional tasks. Participants who had the ability to develop coping strategies appeared to be more resilient and had better mental health. A review by Burke and her colleagues found that individuals focused on physical-psychological factors of CP tended to experience greater fear of pain and depressive symptoms (Burke et al., 2015). However, no studies related to the Arabic population were included in this review. There is an overlap of findings with previous literature about some beliefs about the impact of and coping with CP. However, as discussed in a relatively recent systematic review, they did not investigate these beliefs in light of the future ability to cope (Morton et al., 2019).

Some participants overcame CP-related difficulties by being psychologically resilient, which often involved having a spiritual-faith belief system. This psychological status helped increase acceptance and coping and enhanced behavioural solution-focused approaches. Those participants were generally able to manage their pain and achieve tasks in their daily lives and work despite persistent pain. It is noteworthy that some participants had the ability to use psychological capacities to adapt and recover from the impact of persistent pain. Psychological resilience is considered one of the healthy ways to face difficult circumstances (Newton-John et al., 2014). None of the participants had used psychological interventions or counselling and this seems a missed opportunity. It is worth exploring the availability of culturally sensitive services and any cultural reasons why participants have not accessed these services. Findings revealed that participants relied on social support and spiritual-faith systems for coping with their CP, which can be attributed to the cultural-specific characteristics of the Arabic population. A systematic review by Zolezzi and colleagues showed that stigma towards mental illness treatments is prevalent among patients, care providers and the general public in the Arabic population (Zolezzi et al., 2018). In a Cochrane database review, there were 75 randomised control trials that included psychotherapies for the CP population, yet none involved the Arabic population (de c Williams et al., 2020).

The perception of the available treatments varied among the participants, some of whom had tried numerous different treatments and indicated that the treatments were temporary and did not cure the pain. Some participants did not seem to benefit from any of the available treatments. These findings are consistent with a previous systematic review that explored different treatments for CP and showed that a multidisciplinary treatment approach might have a higher chance of success compared to standard medical treatment (Scascighini et al., 2008). However, the pessimistic view of some of the participants prevented them from trying different alternatives. Further, it seems that some participants had misconceptions about the safety of medications, surgeries, and therapeutic exercises as part of physiotherapy (Taylor et al., 2007; Sanchez and Zurke, 2016), which raise concerns about the quality of services, or the quality of the information provided. The participants’ high expectations led to their entrapment in the pursuit of a cure and perfect outcomes, despite the chronic nature of CP. The participants provided several suggestions for managing their condition on a daily basis, such as the crucial role of willpower. Ridson and colleagues explained how willpower is linked with improved coping mechanisms and increased tolerance towards CP (Risdon et al., 2003). Additionally, some participants mentioned the importance of establishing policies and improving health services for CP.

Despite its several strengths, this study also has important limitations. First, the sample size was shaped by the linked experimental study and did not depend only on the saturation level of the qualitative data study, resulting in repeated data. Second, despite offering breaks before the interviews, participants had to do the interview after the experiment, which they might have found tiring. Third, because this study is concerned with the Arabic population, the ability for transferability and generalisability is limited, and exploring CP-attention experiences in the experimental context for other cultures is encouraged. On the other hand, an advantage was that experiencing the task prompted participants to talk more about attention. Another advantage was the prompt questions in the interview that helped build up a rapport. Further, this study succeeded in unfolding the factors associated with CP maintenance, especially those related to the CP-attention relationship. To further advance the findings of this study, future research is recommended to explore in greater detail the policy changes and enhancements in healthcare services required to address the needs of individuals with CP and their attention difficulties.

## 5. Conclusion

This is the first qualitative study to explore the subjective attention experiences and related impacts of Arabs with CP in both experimental settings and daily attentional tasks. We found that individuals’ everyday functions, including attention, are reciprocally affected by CP symptoms. Participants reported that different factors contribute toward developing or exacerbating CP and affect psychological functions. A positive perception of social support can be essential in managing and coping with CP-related symptoms. Appropriate coping strategies and social support can ameliorate the adverse effects of CP on people’s lives, including attentional difficulties. Participants tried various treatment interventions; however, none used psychological approaches to manage their CP. Based on patient-led suggestions, there is a clear need for improved policies and healthcare services for individuals with CP. The study’s findings provide valuable insights for researchers, practitioners, and policymakers seeking to enhance the well-being of people with CP.

## Data availability statement

The raw data supporting the conclusions of this article will be made available by the authors, without undue reservation.

## Ethics statement

The studies involving humans were approved by the University of Manchester Research Ethics Committee number 3 (UREC 3). The affiliation of UREC 3 is the University of Manchester. The studies were conducted in accordance with the local legislation and institutional requirements. The participants provided their written informed consent to participate in this study.

## Author contributions

AA: Data curation, Formal analysis, Investigation, Methodology, Writing – original draft, Writing – review & editing, Conceptualization. KA: Data curation, Investigation, Writing – review & editing. TK: Data curation, Formal analysis, Methodology, Supervision, Writing – review & editing. MP: Supervision, Writing – review & editing. EP: Supervision, Writing – review & editing. NM: Writing – review & editing. NH: Supervision, Writing – review & editing.
