# Supplementary material for: Exploring chronic pain related attentional experiences, distress and coping strategies among Arabic-speaking individuals in Jordan and the United Kingdom
Source: Front Psychol. 2023 Oct 2;14:1268179. doi: 10.3389/fpsyg.2023.1268179 (PMC10577280; doi:10.3389/fpsyg.2023.1268179)
Supplement: Supplementary file 1 [file Data_Sheet_1.docx]

**Appendix 1: Interview Topic Guide**

**Title: Exploring Chronic Pain-Related Attentional Experiences, Distress and Coping Strategies Among Arabic-Speaking Individuals in Jordan and The United Kingdom**

**Introduction**

- Thank you for seeing me today and offering to participate in this study.
- Explaining a general overview of the project (brief introduction)
- Verbal consent will be taken and recorded separately from the interview.
- I have a list of questions that I want to address your thoughts and feelings about, if possible.
- Feel free to ask questions at any stage during the interview. You can withdraw at any stage if you are feeling uncomfortable.
- I might make a few notes in case I want to come back to something later, and as mentioned in the consent form, this interview will be audio recorded if that is okay for you.
- This interview is confidential; however, confidentiality will be broken if there is a risk of harming the participant or others.

**Topics** (objective aims) of the qualitative semistructured interviews:

1. Identify and elicit details on the experience of participating in experimental research.
2. Identify and elicit details on how the daily attentional experiences are affected due to chronic pain.
3. Identify and elicit details on participants’ perspectives about coping with chronic pain.
4. Explore opinions about possible interventions that could be of benefit from the participant’s perspective.

**Questions**

1. **The experience of participating in experimental research**
   - How did you know about this research? / Who approached you to patriciate?
   - What did you feel/think about participating in this study? (if the participant gave a short answer, ask: Can you tell me more about this? (
   - How did you feel while doing the experimental tasks?
   - What kinds of things might you expect to see in the research that includes the process of assessing attention in people with chronic pain (CP)?
   - How did you feel about responding to pain-related information?
   - What was good about being involved in research/what did you like most/what could make you comfortable?
   - What was not so good about being involved in research/how we can improve /have you faced any difficulties?
   - How would you feel about participating in another study?
   1. **Coping with chronic pain**

- Can you tell me a bit about your chronic pain experiences?
- How did it start and progress?
- Do you have any other medical situation linked to your attention or CP? If yes, could you tell me a bit about it?
- How does CP affect your life?
- What challenges did you face due to chronic pain, if any?
- What do you think about the stress level role in CP exaggeration?
- How do you cope with CP daily, including ADL?
- Do you think your environment's level of social support (family, friends and other critical communities) is linked with your CP situation? If yes, how?

1. **Details on the daily attentional experiences**

- Do you think that chronic pain is affecting your attention ability? (If yes, to which extension your attention problem affects your everyday functionality?
- How is chronic pain impacting your ability to focus?
- How do you feel about the attentional difficulties you face daily?
- How is stress level affecting your attention level?
- How do you cope with attentional difficulties resulting from CP?
  - How do you see the attention role in ameliorating/exaggerating your CP?

1. **Explore opinions about possible interventions.**

-What treatment(s) did you try before to overcome CP?

--What is your opinion about possible treatments/management for your CP?

-What are the treatment-related resources that could be of benefit in managing/coping with your CP?

-What are your expectations regarding CP management?

1. Is there anything else that you (the interviewee) feels has been missed and anything that you did not get a chance to discuss fully/Any other suggestions?

End of interview – thank you.
